# Free-Choice Feeding of Whole Grains Improves Meat Quality and Intestinal Development of Pigeon Squabs Compared with Complete Pelleted Feed

**DOI:** 10.3390/life13030848

**Published:** 2023-03-21

**Authors:** Tingwu Liu, Liuxiong Wang, Xiaoyun Jiang, Ying Liu, Enjie Diao, Peng Xie

**Affiliations:** 1 College of Life Science, Huaiyin Normal University, Huaian 223300, China; 2 College of Animal Science and Technology, Yangzhou University, Yangzhou 225003, China

**Keywords:** pigeon, whole grain, free-choice feeding, meat quality, intestinal development

## Abstract

Effects of different feeding strategies on meat quality and intestinal development in pigeon squabs were investigated. 120 pairs of pigeons with two squabs each were assigned to five groups (four free-choice feeding systems and one complete feeding system): T1 (corn, pea, wheat, and pelleted feed), T2 (corn, wheat, and pelleted feed), T3 (corn, pea, and pelleted feed), T4 (corn and pelleted feed), and T5 (complete pelleted feed). Compared with T5, the diet in T4 made the breast meat redder and more yellow (*p* < 0.05). T2 and T4 resulted in an enhanced total superoxide dismutase activity of meat. Breast muscle in T1 and T2 was determined to have higher contents of nonessential amino acids, glycine, alanine, and glutamic acid (*p* < 0.05). The contents of the essential amino acids, lysine, threonine, valine, histidine, and arginine were also higher in T1 (*p* < 0.05). Villus height, surface area, and alkaline phosphatase activity of the duodenum and jejunum in T2 were the highest among the treatments (*p* < 0.05). In conclusion, free-choice feeding system can improve the meat quality and intestinal development of pigeon squabs, but which combination method of whole grains to use in the production depends on the feeding purpose.

## 1. Introduction

Whole grain (WG) inclusion has gained prominence as a feeding strategy, especially in free-range farming for organic poultry production [1]. Evidence has indicate that WG feeding could decrease the incidence of coccidiosis and *Salmonella enteritidis* infection [2,3], improve the gut microflora ecology [4,5], increase the relative gizzard weight, and promote the apparent metabolizable energy and starch digestibility [6,7]. In addition, further reduction of cost can be achieved due to the simplified handling and processing steps [8]. Free-choice feeding (FCF) is one WG feeding strategy [9]. Since individual birds could have different nutrient requirements for maintenance and production, the ability to self-select feeds to reach their own nutrient targets may not be realized when only one formulated diet is given [1]. 

In Asian countries, raising pigeons for meat has gained popularity in recent years. However, pigeon squabs are reared by their parents for nearly 28 d from hatching, and the whole breeding cycle of pigeon often lasts 2 months, which includes nonbreeding, laying, hatching, and feeding period [10]. The problem is that birds in the same flock are probably in different breeding periods and should have distinct dietary requirements in response to each period. However, a diet formulated to meet all requirements of an average bird during the whole breeding cycle will contain either excessive or inadequate levels of nutrients for individual pigeons above or below the mean. Therefore, FCF system of WG feeding is widely used in the meat-type pigeon production of China, which has been proven to enhance growth performance [11].

Pigeon meat consumption has become popular in more and more countries [12]. Compared with broilers, the muscle of meat-type pigeons is mainly in the breast, and the proportion of thigh is relative lower [11]. The high concentration of protein and the much lower cholesterol found in pigeon muscle make it an ideal candidate for human food resource [13]. Meat quality of animals can be affected by the breed, nutritional level, and feeding management. Positive effects of whole grain feeding on the meat quality in poultry have been reported, which includes improvement of the meat color [14], decreasing the lipid deposition [15], and increasing of trace elements’ content [16]. Commercial organic poultry meat produced by WG feeding is attractive and more expensive for customers in the Chinese market. People also thought that this feeding mode can change the meat flavor because of the improved animal welfare, but the relative knowledge was highly limited. Therefore, the experiment was designed to test the influence of five different feeding strategies (four free-choice feeding systems and one compound feeding system) on meat quality and intestinal development of 28-day-old pigeon squabs.

## 2. Materials and Methods

### 2.1. Animals 

One hundred twenty pairs of 60-week-old American White King pigeons were chosen from Weitekai Pigeon Co., Ltd. (Jiangyin City, China). The birds had the same breeding cycle, and each pair was kept in a cage with a nest and perch. The experiment lasted 35 days, including 7 days of adaptation and 28 days of trial period. 240 pigeon squabs artificially hatched from the incubator were put into the nests when the acclimation was finished, and each pair of parent pigeons only reared two squabs. During the study, water, feed, and sand were supplied *ad libitum*. Sixteen hours of light per day was provided.

### 2.2. Experimental Design and Diets

Pigeons were randomly assigned to five dietary groups, and every group had six replicates with four pairs of adult pigeons (575 ± 25 g) each. Two squabs were kept by each pair of parental pigeons. The treatments are shown in Table 1: T1, corn, pea, wheat, and pelleted feed; T2, corn, wheat, and pelleted feed; T3, corn, pea, and pelleted feed; T4, corn and pelleted feed; and T5, complete pelleted feed. The diet composition is shown in Table 2. The nutritional values were referred to in Xie et al. [11]. The corn, pea, and wheat used in T1–T4 were whole grains. Each whole grain and pelleted feed was offered in separate feeders.

### 2.3. Feed Analysis

The ground pellet diet was prepared for determining the parameters of dry matter (DM, method 925.09), crude protein (CP, method 988.05), crude fat (CF, method 920.39), and ash (method 942.05) according to procedures of AOAC [17]. PARR 1281 oxygen bomb calorimeter (PARR Instrument Co., Moline, IL, USA) was used to measure the gross energy (GE). The nutritional values determined are shown in Table 2.

### 2.4. Sample Collection

As described by our previous study [18], twelve squabs, 28 days of age, from each treatment were sacrificed by cervical dislocation. Weights of empty duodenum, jejunum, and ileum were dividedly recorded, and their organ indexes were calculated as a percentage of body weight. A portion (0.5 cm) of three intestinal segments was collected to be fixed in 10% buffered formalin for histological analysis. Intestinal mucosa were prepared and stored at −80 °C for enzyme assay. The left pectoralis major was sampled and refrigerated at 4 °C for the quality traits test. The right pectoralis major was collected, divided into three parts, and frozen for analyzing proximate composition, antioxidant status, and amino acid profile.

### 2.5. pH, Color, and Water-Holding Capacity

The pH of muscle was analyzed in triplicate by a pH meter (Mettler-Toledo GmBH, Schwerzenbach, Switzerland) at 45 min postmortem. The values of lightness (L*) and redness (a*) of pectoral muscle were measured 45 min postmortem with a Minolta Chroma Meter CR 410 (Minolta Co. Ltd., Osaka, Japan). Shear force, as an index of tenderness of meat, was measured by model C-LM3B meat tenderness meter (Northeast Agricultural University, Harbin, China). The water-holding capacity (WHC) of pigeon meat was determined as described before [19]. Briefly, a 1.0 g meat sample was placed between filter paper and the gravity of 35 kg was used to press it for 5 min; WHC can be calculated as the proportion of water kept in the meat. 

### 2.6. Moisture, Protein, Fat, and Ash Content

The procedures of AOAC [17] were used to analyze the contents of moisture, crude protein, crude fat, and ash in the samples. Meat samples were dried to a constant weight at 105 °C for determining the moisture content. Crude protein content was studied by utilizing an automatic Kjeldahl nitrogen analyzer (Hanon Instruments Co., Ltd., Jinan, China). Crude fat was analyzed by Soxhlet extraction method (SXT-06, Hongji Instruments Co., Ltd., Shanghai, China). For ash content determination, meat samples were incinerated to a constant weight at 550 °C in a muffle furnace. 

### 2.7. Antioxidant Status

As described by Ciftci et al. with some modifications [20], approximately 0.5 g of breast meat were homogenized (1:9, *w*/*v*) in 150 mmol/L NaCl (ice-cold) and centrifuged. The supernatant was used to analyze the activities of glutathione peroxidase (GSH-Px), catalase (CAT), and total superoxide dismutase (T-SOD), as well as the content of malondialdehyde (MDA) using diagnostic kits (Nanjing Jiancheng Bioengineering Institute, Nanjing, China) by spectrophotometry. Coomassie Brilliant Blue G250 was used to measure the total protein content of samples. One unit (1U) of T-SOD can be expressed as the amount of SOD enzyme per milligram of tissue protein reaching the 50% inhibition in 1 mL reaction solution. One µmol of hydrogen peroxide decomposed per second in 1 milligram of tissue protein was defined as one unit of CAT activity. One unit of GSH-Px enzyme was defined as the concentration of GSH reduced by 1 μmol/L in the reaction system every minute in each milligram of tissue protein. 

### 2.8. Amino Acid Profile

As described by Czauderna er al. [21], briefly, 0.05 g of the meat sample was hydrolyzed with 6 M HCl for 24 h at 110 °C. The resolution was diluted to a volume of 50 mL and then filtered and evaporated in a water bath. Amino acid concentrations were determined by an automatic amino acid analyzer (AAA L-8900, Hitachi High-Technologies Co., Ltd., Tokyo, Japan). The following amino acids were evaluated: proline, glutamic acid, glycine, alanine, serine, aspartic acid, cysteine, tyrosine, lysine, methionine, threonine, valine, leucine, isoleucine, histidine, arginine, and phenylalanine. The resultant amino acid values were calculated to a 100% meat sample. 

### 2.9. Gut Histomorphology

Briefly, the gut samples were washed for removing formaldehyde and embedded in paraffin after dehydration. Then, hematoxylin-eosin was used to stain 5 μm-thick slices. Morphometric analyses were performed using a microscope and analyzed with Image J software (version 1.46, NIH, USA). Villus length was measured from the tip of the villus to the crypt junction. Depth of crypts of Lieberkühn was determined from the base upward to the region of transition. The villus surface area was calculated by the formula [22]: villus surface area = (2π) × (villus width/2) × (villus height).

### 2.10. Enzyme Activity

The middle part of the intestine was rinsed in pH 7.4 buffer solution (10 mM Tris succinate, 150 mM mannitol, 1 mM MnCl_2_, and 5 mM K_2_PO_4_, 5 mM MgCl_2_) [23], and then, the mucosal surface was sampled and homogenized in the above-mentioned buffer containing 2% triton X-100 by an Ultra-turrax homogenizer (IKA), and centrifuged for 10 min at 10,000× *g*. The supernatant was used to examine the activities of maltase (MLA), leucine aminopeptidase (LAP), and alkaline phosphatase (AKP) by microplate reader. Activities of MLA and AKP were examined by using the diagnostic kits (Nanjing Jiancheng Bioengineering Institute, Nanjing, China). An ELISA kit (RapidBio Research Lab, West Hills, CA, USA) was used to measure LAP activity. Total protein concentrations were analyzed using the Coomassie Brillant Blue G250 method. Activities of three enzymes were all calculated as units per milligram of sample protein.

### 2.11. Statistical Analysis

All the experimental data were analyzed by SPSS 17.0. ANOVA followed by Tukey’s multiple comparison test was conducted. The differences were judged significant at *p* < 0.05.

## 3. Results

Whole grain feeding can be well accepted by the birds (Table 3). 

Table 4 showed the influence of five different feeding strategies on meat quality of pigeon breast muscle at 28 days-of-age. The values of pH, L*, water-holding capacity, and shear force were not influenced by feeding strategies (*p* > 0.05). a* and b* values in T4 were highest among the five treatments (*p* = 0.038; *p* < 0.001).

As shown in Table 5, no effects of different feeding strategies on the contents of moisture, CP, and ash in pigeon meat were found (*p* > 0.05). The content of CF in breast muscle in T2 was higher than that in T5 (*p* = 0.032).

There was no significant difference in the activity of GSH-Px among different feeding groups (*p* = 0.262) (Table 6). The T-SOD activity of pigeon breast meat in T2 and T4 was higher than that in T5 (*p* = 0.046), and CAT activity in the T5 group was also the lowest (*p* = 0.025). MDA content in pigeon breast meat of T5 tended to increase, but it was not significant (*p* = 0.058).

As shown in Table 7, higher values of the nonessential amino acids, glycine (1.09%), and tyrosine (0.84%), and the essential amino acids, lysine (1.97%), threonine (1.10%), valine (1.09%), histidine (0.67%), and arginine (1.54%) were observed in T1 (*p* < 0.05). The contents of glutamic acid (3.26%), glycine (1.16%), alanine (1.49%), and aspartic acid (2.07%) were also higher in T2. The proline (0.41%) content of pigeon breast meat was higher in T3 (*p* = 0.035). However, complete pelleted feeding resulted in lower levels of the majority of essential amino acids in pigeon breast muscle. 

Organ index of pigeon duodenum, jejunum, and ileum showed no changes under different feeding strategies (*p* > 0.05) (Table 8). However, different feeding systems have significant effects on intestinal histomorphology in 28-day-old squabs (*p* < 0.05) (Table 9). Villus height and surface area of duodenum and jejunum in T2 group were the highest among the treatments and squabs in the group while T5 received complete pelleted diet and had the lowest villus height (*p* < 0.05). Surface area of pigeon jejunum in T1 was lower than other groups (*p* < 0.05). Crypt depth of three intestinal segments had no changes under the different feeding strategies (*p* > 0.05). 

As shown in Table 10, AKP activity in duodenum and jejunum was the highest in 28-day-old squabs reared by parental pigeons fed corn, wheat, and pelleted feed, and it decreased significantly in the pigeon group fed complete pelleted feed (*p* < 0.05). Different feeding strategies showed no significant effects on activity of MLA and LAP in three intestinal segments (*p* > 0.05).

## 4. Discussion

A theory was put forth that the nutrient requirement can be achieved by self-selection of the birds themselves [1]. Only one completed study may not satisfy every specific nutrient requirement of breeding pigeons during different physiological period [11]. Food preference (corn > pea, wheat) of pigeons was reported by Sales and Janssen [24]. Therefore, choice feeding based on unground corn and high-protein pellets gained popularity for a long time. Until now, both WG feeding and complete feeding are applied in pigeon production, but different combinations of corn, pea, wheat, and pelleted feed in the WG feeding strategy were used by different producers. Our previous study found that choice feeding of corn, wheat, and pelleted feed can enhance the growth performance of pigeons and was thought to be commercially available [11]. However, the acceptance of a type of whole grain by birds is mainly attributed to its shape, color, size, and nutrients concentration or, probably, the involvement of all these factors [5]. In the present study, wheat in WG feeding groups seemed to be well-received due to its higher relative intake. Smaller size and convenience for pecking may be the major reason, while corn and pea are large seeds and are not easy to ingest by poultry [25]. 

Animal meat quality was closely related to the feed ingredients [26]. Bright flesh color can stimulate customers’ desire to buy. The values of redness and yellowness in T4 were higher than that in other treatments in our study, and more relative intake of corn ingested by pigeons in T4 may be an important reason. Since corn was found to be a major contributor of dietary natural pigments (zeaxanthin and lutein) [27], the redness of meat is closely related to the oxymyoglobin content [28]. Supplementation of the protein-xanthophylls concentrate of alfalfa made the muscle redder and of a higher oxymyoglobin content [29]. Lutein-supplemented chickens also had greater yellowness in breast meat [30]. Introducing only wheat grain in the diet of fattening turkeys gained the opposition results [31] which can be attributed to the lower intake of natural pigments. 

Vandeputte-Poma [32] reported that the content of grains in crop milk increased steadily after 12 days of age. The inclusion of WG in poultry diet was shown to improve the nutrient uptake and utilization [8,33]. Whole grains in crop milk brought by parent pigeons probably affected the energy utilization of squabs, but different combinations of whole grains (corn, pea, and wheat) may have different feeding effects, which could be attributed to the size or nutritive value of grains. Ye et al. [34] reported that the shear force of breast meat was correlated with intramuscular fat in pigeon squabs, which is similar to that in mammals [35]. However, there was no significant difference in shear force among the five treatments. 

Lipid-derived free radicals can damage the structure of cells through oxidation due to their highly unstable characteristics [36]. Lipids oxidation in meat can lead to a decrease in nutritional and sensory value [37]. Therefore, the antioxidant status, which can be reflected by the activities of antioxidant enzymes (GSH-Px, CAT, and SOD) and the concentration of the end product of lipid oxidation (MDA), is closely linked to meat quality [38]. Previous studies found that free radicals produced by lipid oxidation of meat can get into the cytoplasm, and the reaction with oxymyoglobin and metmyoglobin accumulation can be accelerated, which in turn decreases the value of a* and causes meat discoloration [39,40,41]. These findings can explain the relatively lower value of redness and yellowness of breast meat in T5. Lutein and zeaxanthin in corn are important antioxidant carotenoids [42]. The inclusion of whole grain of corn in the treatments, especially in T4, can bring more content of carotenoids, which can decrease oxidative damage in poultry [30,43].

Amino acids are important sources of aroma flavor, which are involved in the Maillard reaction and Strecker degradation [44]. Different amino acids elicit different tastes; for example, phenylalanine, histidine, tyrosine, methionine, valine, and arginine are bitter in taste, whereas glutamic acid is sour and umami in taste, and proline elicits both sweet and bitter tastes [45]. Glutamic acid, alanine, glycine, and arginine are also important precursors of meat flavor volatiles [46]. It was reported that duck muscle under the whole wheat feeding system contained more glutamic acid, alanine, and arginine [47], especially glutamic acid, which may be due to its higher content in dietary wheat compared to that in corn. Digestibility of ileal protein and concentrations of alanine, asparagine, and cysteine in systemic plasma can be effectively increased by WG feeding [48,49]. The whole grains appeared in crop milk probably affected amino acid deposition in squabs’ breast meat. In the present study, both essential amino acids (lysine, threonine, valine, histidine, and arginine) and nonessential amino acids (glutamic acid, glycine, alanine, and tyrosine) measured in breast muscle in T1 were higher than in T5, possibly indicating the greater nutritive value in meat. However, different grain types or combinations in diets may exert different effects on the same animal species, and further research is warranted.

Other than the hyperplasia of the gizzard organ, relative weight of intestinal segments in birds fed a diet with whole grains inclusion commonly shows no changes in most studies [2,33,50]. This is consistent with our present study results. Although the research investigating the effect of WG feeding on animal gastrointestinal morphology were limited, controversial results were reported. Gabriel et al. [51] found that villus surface area and the length of villus to crypt increased significantly in the duodenum of whole wheat-fed broilers at 23 days of age. Meanwhile, no influence of whole grain feeding on intestinal morphology was also reported [33]. Generally, the longer and larger intestinal villi are beneficial for the digestion and absorption of nutrients [52]. The form and composition of diet would change from the upper part to the lower part of the intestine. The morphology of the upper small intestine was expected to improve when poultry was fed a diet with whole grains inclusion [53]. The present study found that the choice feeding of corn, wheat, and pelleted feed increased villus length and surface area of the upper small intestine in squabs compared with that in the complete pelleted feed group, which showed small intestines structurally more oriented to digestion. Interestingly, not all whole grains feeding treatments showed such a positive effect. Previous studies reported that histological changes are more likely to be induced by the content and physicochemical properties of dietary fiber other than the protein intake [54,55,56]. Therefore, different combinations of whole grains may bring a different intake of dietary fiber, which in turn affected the pigeon intestinal morphology. 

Studies investigating the effect of whole grain feeding on digestive enzyme activity were also scarce. AKP is considered to be a marker for enterocyte maturity, which can account for the migration of epithelial cells, and finally affecting animal villus morphology [57]. In pigeon duodenum and jejunum and the higher villus height of WG feeding treatment was associated with a higher AKP activity in the present study, which further confirmed the above viewpoint. MLS and LAP are responsible for nutrient digestion [58], and they were also thought to be enterocyte maturation marker for digestive function in growing pigeons [59]. However, activities of these two enzymes in our study showed no changes under the different feeding strategies. It indicated that choice feeding of whole grains probably had little effect on intestinal digestive function.

## 5. Conclusions

Using the choice feeding strategy of corn and pelleted feed made the breast meat redder and more yellow and enhanced the muscular antioxidant capacity. However, the feeding strategy of whole grains of corn, pea, wheat, and pelleted feed increased the contents of both nonessential and essential amino acids in breast meat, which could affect the flavor and nutritive value of meat. Morphology of duodenum and jejunum were improved in squabs on a choice feeding of corn, wheat, and pelleted feed. However, which whole grain combination method would be used in production depends on the feeding purpose.

## Figures and Tables

**Table 1:** Five different feeding strategies used in the present experiment.

Item	Diet ^1^				
	T1	T2	T3	T4	T5
Corn	+	+	+	+	-
Pea	+	-	+		-
Wheat	+	+	-	-	-
Concentrate feed	+	+	+	+	-
Compound feed	-	-	-	-	+

^1^ T1, corn, pea, wheat, and pelleted feed; T2, corn, wheat, and pelleted feed; T3, corn, pea, and pelleted feed; T4, corn and pelleted feed; T5, complete pelleted feed.

**Table 2:** Ingredients and nutrient composition of the diets.

Item	Pelleted Feed	Complete Pelleted Feed
Ingredient (g/kg)		
Yellow corn	460	550
Soybean meal, 44.2% CP	350	245
Wheat	100	110
Soybean oil	26	20
Limestone	30	20
Dicalcium phosphate	16	12
Sodium chloride	4	2.5
Premix ^1^	8	5
Zeolite powder	3.2	34.2
Lysine	1	0.7
Methionine	1.8	0.6
Determined analysis		
Gross Energy (MJ/kg)	16.42	16.59
Dry matter (%)	86.83	87.15
Crude protein (%)	20.36	16.52
Crude fat (%)	5.33	4.67
Ash (%)	6.45	8.01
Calculated concentration		
Metabolic Energy (MJ/kg)	11.88	12.00
Crude protein (%)	20.50	16.67
Lysine (%)	1.17	0.89
Methionine (%)	0.48	0.31
Calcium (%)	1.65	1.13
Available P (%)	0.43	0.34

^1^ Each kg contains: Vit. A, 2.2 mg; Vit. D3, 0.043 mg; Vit. E, 24 mg; Vit. K3, 1 mg; Vit. B1, 3 mg; Vit. B2, 13 mg; Vit. B6, 2 mg; Vit. B12, 2.5 mg; niacin, 15 mg; pantothenic acid, 7.5 mg; folic acid, 0.55 mg; biotin, 0.12 mg; choline chloride, 200 mg; iron, 35 mg; manganese, 55 mg; copper, 10 mg; iodine, 0.2 mg; zinc, 35 mg; selenium, 0.25 mg.

**Table 3:** Relative intake of parent pigeons under different feeding strategies ^1^.

Item	Relative Intake (%)				
	T1	T2	T3	T4	T5
Corn	25.4	18.9	37.1	56.3	
Pea	24.2		30.1		
Wheat	29.5	47.1			
Pelleted feed	20.9	34.0	32.8	43.7	
Complete pelleted feed					100

^1^ T1, corn, pea, wheat, and pelleted feed; T2, corn, wheat, and pelleted feed; T3, corn, pea, and pelleted feed; T4, corn and pelleted feed; T5, complete pelleted feed.

**Table 4:** Meat quality parameters in pigeon breast meat under different feeding strategies.

Item	Diet ^1^					SEM	*p*-Value
	T1	T2	T3	T4	T5		
pH	5.82	5.83	5.87	5.82	5.79	0.022	0.850
Redness (a*)	11.49 ^ab^	11.37 ^ab^	11.51 ^ab^	11.88 ^a^	10.96 ^b^	0.10	0.038
Yellowness (b*)	9.42 ^a^	9.45 ^a^	9.68 ^a^	9.96 ^a^	8.19 ^b^	0.11	<0.001
Lightness (L*)	36.79	37.42	37.13	37.71	36.55	0.17	0.165
Water-holding capacity (%)	74.29	76.27	75.72	76.98	73.05	0.60	0.235
Shear force (kgf)	1.60	1.66	1.61	1.60	1.51	0.042	0.804

^1^ T1, corn, pea, wheat, and pelleted feed; T2, corn, wheat, and pelleted feed; T3, corn, pea, and pelleted feed; T4, corn and pelleted feed; T5, complete pelleted feed. ^a,b^ Different letters mean significant difference (*p* < 0.05).

**Table 5:** Chemical contents in pigeon breast meat under different feeding strategies.

Item	Diet ^1^					SEM	*p*-Value
	T1	T2	T3	T4	T5		
Moisture (%)	74.22	74.13	74.01	73.82	73.75	0.14	0.668
Crude protein (%)	21.11	21.20	21.03	21.18	20.96	0.12	0.928
Crude fat (%)	3.33 ^ab^	3.67 ^a^	3.35 ^ab^	3.40 ^ab^	3.10 ^b^	0.059	0.032
Ash (%)	0.78	0.76	0.77	0.82	0.79	0.0054	0.215

^1^ T1, corn, pea, wheat, and pelleted feed; T2, corn, wheat, and pelleted feed; T3, corn, pea, and pelleted feed; T4, corn and pelleted feed; T5, complete pelleted feed. ^a,b^ Different letters mean significant differences (*p* < 0.05).

**Table 6:** Antioxidant capacity of pigeon breast meat under different feeding strategies.

Item ^2^	Diet ^1^					SEM	*p*-Value
	T1	T2	T3	T4	T5		
GSH-Px (U/mg protein)	22.88	21.65	27.76	22.012	21.34	0.99	0.262
CAT (U/g protein)	0.41 ^ab^	0.39 ^ab^	0.49 ^ab^	0.52 ^a^	0.33 ^b^	0.022	0.025
T-SOD (U/mg protein)	60.06 ^ab^	64.06 ^a^	58.89 ^ab^	63.99 ^a^	52.01 ^b^	1.55	0.046
MDA (mmol/g protein)	0.69	0.68	0.78	0.65	0.83	0.025	0.058

^1^ T1, corn, pea, wheat, and pelleted feed; T2, corn, wheat, and pelleted feed; T3, corn, pea, and pelleted feed; T4, corn and pelleted feed; T5, complete pelleted feed. ^2^ GSH-Px, glutathione peroxidase; CAT, catalase; T-SOD, total superoxide dismutase; MDA, malondialdehyde. ^a,b^ Different letters mean significant differences (*p* < 0.05).

**Table 7:** Amino acid profile of pigeon breast muscle under different feeding strategies.

Item	Diet ^1^					SEM	*p*-Value
	T1	T2	T3	T4	T5		
Nonessential amino acid							
Proline	0.36 ^ab^	0.40 ^ab^	0.41 ^a^	0.33 ^b^	0.36 ^ab^	0.011	0.035
Glutamic acid	3.18 ^a^	3.26 ^a^	2.92 ^ab^	2.80 ^b^	3.18 ^a^	0.058	<0.001
Glycine	1.09 ^a^	1.16 ^a^	0.95 ^b^	0.91 ^b^	0.94 ^b^	0.023	<0.001
Alanine	1.46 ^ab^	1.49 ^a^	1.31 ^bc^	1.30 ^c^	1.29 ^c^	0.023	<0.001
Serine	0.73	0.74	0.80	0.77	0.75	0.010	0.257
Aspartic acid	2.02 ^ab^	2.07 ^a^	1.94 ^ab^	1.95 ^ab^	1.92 ^b^	0.020	0.048
Cysteine	0.22	0.21	0.20	0.20	0.20	0.004	0.414
Tyrosine	0.84 ^a^	0.79 ^ab^	0.75 ^ab^	0.72 ^b^	0.71 ^b^	0.014	0.014
Essential amino acid							
Lysine	1.97 ^a^	1.84 ^ab^	1.91 ^ab^	1.81 ^b^	1.80 ^b^	0.020	0.010
Methionine	0.56	0.52	0.57	0.59	0.56	0.009	0.192
Threonine	1.10 ^a^	1.00 ^ab^	1.09 ^ab^	1.00 ^ab^	0.99 ^b^	0.081	0.041
Valine	1.09 ^a^	1.01 ^ab^	1.08 ^ab^	1.00 ^ab^	0.99 ^b^	0.014	0.035
Leucine	1.95	1.86	1.91	1.83	1.82	0.020	0.139
Isoleucine	0.98	0.92	0.95	0.91	0.89	0.013	0.127
Histidine	0.67 ^a^	0.61 ^ab^	0.65 ^ab^	0.60 ^ab^	0.58 ^b^	0.011	0.046
Arginine	1.54 ^a^	1.37 ^ab^	1.49 ^ab^	1.35 ^b^	1.35 ^b^	0.023	0.008
Phenylalanine	0.94	0.96	0.93	0.92	0.92	0.012	0.896

^1^ T1, corn, pea, wheat, and pelleted feed; T2, corn, wheat, and pelleted feed; T3, corn, pea, and pelleted feed; T4, corn and pelleted feed; T5, complete pelleted feed. ^a–c^ Different letters mean significant differences (*p* < 0.05).

**Table 8:** Organ index of small intestine in pigeon squabs under different feeding strategies.

Item	Diet ^1^					SEM	*p*-Value
	T1	T2	T3	T4	T5		
Organ index (%)							
Duodenum	0.83	0.90	0.84	0.82	0.84	0.022	0.552
Jejunum	1.26	1.30	1.24	1.22	1.21	0.025	0.773
Ileum	0.95	1.19	1.10	1.05	1.06	0.041	0.267

^1^ T1, corn, pea, wheat, and pelleted feed; T2, corn, wheat, and pelleted feed; T3, corn, pea, and pelleted feed; T4, corn and pelleted feed; T5, complete pelleted feed.

**Table 9:** Morphology of small intestine in pigeon squabs under different feeding strategies.

Item	Diet ^1^					SEM	*p*-Value
	T1	T2	T3	T4	T5		
Villus height (μm)							
Duodenum	1659.15 ^bc^	2079.44 ^a^	1834.70 ^ab^	1716.40 ^b^	1541.99 ^c^	43.59	<0.001
Jejunum	1212.65 ^ab^	1300.43 ^a^	1243.48 ^ab^	1251.76 ^ab^	1149.17 ^b^	19.64	0.018
Ileum	843.20	814.23	753.57	688.27	837.50	18.01	0.067
Crypt depth (μm)							
Duodenum	178.08	184.79	164.47	187.11	177.65	3.50	0.088
Jejunum	124.71	134.34	129.05	118.55	127.46	1.97	0.079
Ileum	85.04	92.81	88.07	80.19	82.66	2.11	0.561
Surface area (mm2)							
Duodenum	1.98 ^ab^	2.79 ^a^	2.18 ^ab^	1.94 ^ab^	1.88 ^b^	0.12	0.016
Jejunum	1.25 ^b^	1.74 ^a^	1.69 ^a^	1.54 ^ab^	1.36 ^ab^	0.09	0.045
Ileum	0.88	0.85	0.72	0.78	0.68	0.04	0.261

^1^ T1, corn, pea, wheat, and pelleted feed; T2, corn, wheat, and pelleted feed; T3, corn, pea, and pelleted feed; T4, corn and pelleted feed; T5, complete pelleted feed. ^a–c^ Different letters mean significant difference (*p* < 0.05).

**Table 10:** Activities of enzyme in mucosal homogenates of pigeon intestine under different feeding strategies.

Item ^2^	Diet ^1^					SEM	*p*-Value
	T1	T2	T3	T4	T5		
Duodenum (U/g of protein)							
MLA	0.84	0.72	0.67	0.84	0.79	0.05	0.369
LAP	1.71	2.01	1.89	1.54	1.95	0.16	0.517
AKP	2521 ^b^	4204 ^a^	3580 ^a^	1902 ^b^	3106 ^ab^	319	<0.001
Jejunum (U/g of protein)							
MLA	0.27	0.29	0.29	0.31	0.24	0.02	0.870
LAP	2.08	2.58	2.37	2.77	2.43	0.19	0.630
AKP	1014 ^a^	1093 ^a^	559 ^b^	481 ^b^	626 ^b^	60	0.015
Ileum (U/g of protein)							
MLA	0.18	0.17	0.16	0.15	0.15	0.01	0.778
LAP	2.62	3.15	2.49	3.08	2.82	0.20	0.378
AKP	429	531	332	457	352	37	0.406

^1^ T1, corn, pea, wheat, and pelleted feed; T2, corn, wheat, and pelleted feed; T3, corn, pea, and pelleted feed; T4, corn and pelleted feed; T5, complete pelleted feed. ^2^ MLA: maltase; AKP: alkaline phosphatase; LAP: leucine aminopeptidase. ^a,b^ Different letters mean significant differences (*p* < 0.05).

## Data Availability

The data that support their conclusions are available from the authors of this manuscript upon request.

## References

[1] Fanatico A.C., Brewer V.B., Owens-Hanning C.M., Donoghue D.J. (2013). Free-choice feeding of free-range meat chickens. J. Appl. Poult. Res..

[2] Banfield M.J., Kwakkel R.P., Forbe J.M. (2002). Effects of wheat structure and viscosity on coccidiosis in broiler chickens. Anim. Feed Sci. Technol..

[3] Ratert C., Sander S.J., Verspohl J., Beyerbach M., Kamphues J. (2015). Effects of the physical form of diet on the outcome of an artificial *Salmonella* infection in broilers. Avian Dis..

[4] Gabriel I., Mallet S., Leconte M. (2003). Differences in the digestive tract characteristics of broiler chickens fed on complete pelleted diet or on whole wheat added to pelleted protein concentrate. Brit. Poult. Sci..

[5] Singh Y., Molan A.L., Ravindran V. (2019). Influence of the method of whole wheat inclusion on performance and caecal microbiota profile of broiler chickens. J. Appl. Anim. Nutri..

[6] Santos F.B.O., Sheldon B.W., Santos A.A., Ferket P.R. (2008). Influence of housing system, grain type, and particle size on *Salmonella* colonization and shedding of broilers fed triticale or corn-soybean meal diets. Poult. Sci..

[7] Truong H.H., Moss A.F., Liu S.Y., Selle P.H. (2017). Pre- and post-pellet whole grain inclusions enhance feed conversion efficiency, energy utilisation and gut integrity in broiler chickens offered wheat-based diets. Anim. Feed Sci. Technol..

[8] Svihus B., Juvik E., Hetland H., Krogdahl Å. (2004). Causes for improvement in nutritive value of broiler chicken diets with whole wheat instead of ground wheat. Brit. Poult. Sci..

[9] Fanatico A.C., Owenshanning C.M., Gunsaulis V.B., Donoghue A.M. (2016). Choice feeding of protein concentrate and grain to organic meat chickens. J. Appl. Poult. Res..

[10] Dong X.Y., Zhang M., Jia Y.X., Zou X.T. (2013). Physiological and hormonal aspects in female domestic pigeons (*Columba livia*) associated with breeding stage and experience. J. Anim. Physiol. Anim. Nutr..

[11] Xie P., Jiang X.Y., Bu Z., Fu S.Y., Zhang S.Y., Tang Q.P. (2016). Free choice feeding of whole grains in meat-type pigeons: 1. effect on performance, carcass traits and organ development. Brit. Poult. Sci..

[12] Zieleziński M., Pawlina E. (2005). Użytkowanie mięsne gołębi (Pigeon breeding for meat). Pol. Drob..

[13] Pomianowski J.F., Mikulski D., Pudyszak K., Cooper R.G., Angowski M., Jozwik A., Horbanczuk J.O. (2009). Chemical composition, cholesterol content, and fatty acid profile of pigeon meat as influenced by meat-type breeds. Poult. Sci..

[14] Zhang R., Ma H., Han P., Li Y., Sun Y., Yuan J., Wang Y., Ni A., Zong Y., Bian S. (2022). Effects of feed systems on growth performance, carcass characteristics, organ index, and serum biochemical parameters of pigeon. Poult. Sci..

[15] Amerah A.M., Ravindran V. (2008). Influence of method of whole-wheat feeding on the performance, digestive tract development and carcass traits of broiler chickens. Anim. Feed Sci. Technol..

[16] Song Y., Li Y., Zheng S., Dai W., Shen X., Zhang Y., Zhao W., Chang G., Xu Q., Chen G. (2017). Effects of forage feeding versus grain feeding on the growth performance and meat quality of Yangzhou geese. Brit. Poult. Sci..

[17] Association of Official Analytical Chemists [AOAC] (1990). Official Methods of Analysis.

[18] Xie P., Wang Y., Wang C., Yuan C., Zou X.T. (2013). Effect of different fat sources in parental diets on growth performance, villus morphology, digestive enzymes and colorectal microbiota in pigeon squabs. Arch. Anim. Nutr..

[19] Jiang Z.Y., Jiang S.Q., Lin Y.C., Xi P.B., Yu D.Q., Wu T.X. (2007). Effects of soybean isoflavone on growth performance, meat quality, and antioxidation in male broilers. Poult. Sci..

[20] Ciftci O., Ozdemir İ., Aydin M., Beytur A. (2012). Beneficial effects of chrysin on the reproductive system of adult male rats. Andrologia.

[21] Czauderna M., Kowalczyk J., Niedzwiedzka K.M., Wasowska I. (2002). Determination of free- and protein primary amino acids in biological materials by high-performance liquid chromatography and photodiode array detection. J. Anim. Feed Sci..

[22] Sakamoto K., Hirose H., Onizuka A., Hayashi M., Futamura N., Kawamura Y., Ezaki T. (2000). Quantitative study of changes in intestinal morphology and mucus gel on total parenteral nutrition in rats. J. Surg. Res..

[23] Jang I.S., Ko Y.H., Kang S.Y., Lee C.Y. (2007). Effect of a commercial essential oil on growth performance, digestive enzyme activity and intestinal microflora population in broiler chickens. Anim. Feed Sci. Technol..

[24] Sales J., Janssens G.P.J. (2002). Nutrition of the domestic pigeon (*Columba livia domestica*). World’s Poult. Sci..

[25] Adret-Hausberger M., Cumming R.B., Farrell D.J. (1985). Behavioural aspects of food selection in young chickens. Recent Advances in Animal Nutrition in Australia.

[26] Lebret B., Prache S., Berri C., Lefèvre F., Alami-Durante H. (2015). Meat quality: Influence of animals’ characteristics and rearing conditions. INRA Prod. Anim..

[27] Alisa P., Helen R., Elizabethj J. (2009). Xanthophyll (lutein, zeaxanthin) content in fruits, vegetables and corn and egg products. J. Food Compos. Anal..

[28] Renerre M., Dumont F., Gatellier P. (1996). Antioxidant enzyme activities in beef in relation to oxidation of lipid and myoglobin. Meat Sci..

[29] Karwowska M., Stadnik J., Dolatowski Z.J., Grela E.R. (2010). Effect of protein-xanthophylls (px) concentrate of alfalfa supplementation on physico-chemical properties of turkey breast and thigh muscles during ageing. Meat Sci..

[30] Rajput N., Naeem M., Ali S., Zhang J.F., Zhang L., Wang T. (2013). The effect of dietary supplementation with the natural carotenoids curcumin and lutein on broiler pigmentation and immunity. Poult. Sci..

[31] Majewska T. (1995). Whole wheat grain in young slaughter turkey feeding. Acta Acad. Agric. Tech. Olst..

[32] Vandeputte-Poma J. (1980). Feeding, growth and metabolism of the pigeon, *Columba livia domestica*: Duration and role of crop milk feeding. J. Comp. Physiol. B..

[33] Wu Y.B., Ravindran V., Thomas D.G., Birtles M.J., Hendriks W.H. (2004). Influence of method of whole wheat inclusion and xylanase supplementation on the performance, apparent metabolisable energy, digestive tract measurements and gut morphology of broilers. Brit. Poult. Sci..

[34] Ye M.H., Zhou B., Wei S.S., Ding M.M., Lu X.H., Shi X.H., Ding J.T., Yang S.M., Wei W.H. (2016). Transcriptomic analysis identifies candidate genes related to intramuscular fat deposition and fatty acid composition in the breast muscle of squabs (*Columba*). G3 Genes.

[35] Jeleníková J., Pipek P., Miyahara M. (2008). The effects of breed, sex, intramuscular fat and ultimate pH on pork tenderness. Eur. Food Res. Technol..

[36] Sreejayan N., Rao M.N. (1996). Free radical scavenging activity of curcuminoids. Arzneimittel-Forsch.

[37] Manhiani P.S., Northcutt J.K., Han I., Bridges W.C., Dawson P.L. (2013). Antioxidant activity of carnosine extracted from various poultry tissues. Poult. Sci..

[38] Utama D.T., Lee S.G., Baek K.H., Kim H.K., Lee S.K. (2016). Correlation between antioxidant enzyme activity, free iron content and lipid oxidation in four lines of Korean native chicken meat. Korean J. Food Sci. Anim. Resour..

[39] Schaefer D.M., Liu Q., Faustman C., Yin M.C. (1995). Supranutritional administration of vitamins E and C improves oxidative stability of beef. J. Nutr..

[40] Faustman C., Sun Q., Mancini R., Suman S.P. (2010). Myoglobin and lipid oxidation interactions: Mechanistic bases and control. Meat Sci..

[41] Zhang W., Xiao S., Lee E.J., Ahn D.U. (2010). Consumption of oxidized oil increases oxidative stress in broilers and affects the quality of breast meat. J. Agric. Food. Chem..

[42] Jiao Y., Li D., Chang Y., Xiao Y. (2018). Effect of freeze-thaw pretreatment on extraction yield and antioxidant bioactivity of corn carotenoids (lutein and zeaxanthin). J. Food Qual..

[43] Shanmugasundaram R., Selvaraj R.K. (2011). Lutein supple mentation alters inflammatory cytokine production and antioxidant status in F-line turkeys. Poult. Sci..

[44] Virgili R., Saccani G., Gabba L., Tanzi E., Bordini C.S. (2007). Changes of free amino acids and biogenic amines during extended ageing of Italian dry-cured ham. LWT-Food Sci. Technol..

[45] Kato H., Rhue M.R., Nishimura T. (1989). Role of free amino acids and peptides in food taste. ACS Symposium Series.

[46] Lee S.M., Kwon G.Y., Kim K.O., Kim Y.S. (2011). Metabolomic approach for determination of key volatile compounds related to beef flavor in glutathione-Maillard reaction products. Anal. Chim. Acta.

[47] Kokoszynski D., Kotowicz M., Brudnicki A., Bernacki Z., Wasilewski P.D., Wasilewski R. (2016). Carcass composition and quality of meat from Pekin ducks finished on diets with varying levels of whole wheat grain. Anim. Prod. Sci..

[48] Amerah A.M., Peron A., Zaefarian F., Ravindran V. (2012). Influence of whole wheat inclusion and a blend of essential oils on the performance, nutrient utilisation, digestive tract development and ileal microbiota profile of broiler chickens. Brit. Poult. Sci..

[49] Yin D., Chrystal P.V., Moss A.F., Liu S.Y., Yuan J., Selle P.H. (2020). Effects of reducing dietary crude protein and whole grain feeding on performance and amino acid metabolism in broiler chickens offered wheat-based diets. Anim. Feed Sci. Technol..

[50] Ravindran V., Wu Y.B., Thomas D.G., Morel P.C.H. (2006). Influence of whole wheat feeding on the development of gastrointestinal tract and performance of broiler chickens. Aust. J. Agric. Res..

[51] Gabriel I., Mallet S., Leconte M., Travel A., Lalles J.P. (2008). Effect of whole wheat feeding on the digestive tract of broiler chickens. Anim. Feed Sci. Technol..

[52] Onderci M., Sahin N., Sahin K., Cikim G., Aydin A., Ozercan I., Aydin S. (2006). Efficiency of supplementation of -amylase-producing bacteria culture on the performance, nutrient use and gut morphology of broiler chickens fed a corn-based diet. Poult. Sci..

[53] Mahdavi Sadati M.S., Rezaeipour V., Abdullahpour R. (2022). Efficacy of whole wheat grain in combination with acidified drinking water on growth performance, gizzard development, intestinal morphology, and microbial population of broiler chickens. Livest. Sci..

[54] Jankowski J., Juskiewicz J., Gulewicz K., Lecewicz A., Slominski B.A., Zduńczyk Z. (2009). The effect of diets containing soybean meal, soybean protein concentrate, and soybean protein isolate of different oligosaccharide content on growth performance and gut function of young turkeys. Poult. Sci..

[55] Juskiewicz J., Jankowski J., Zdunczyk Z., Lecewicz A., Przybylska-Gornowicz B., Zieba M. (2009). Effect of diets with different contents of soybean α-galactosides and crude fibre on modification of duodenal microstructure and selected parameters of nutrient utilization in young turkeys. Pol. J. Vet. Sci..

[56] Buwjoom T., Yamauchi K., Erikawa T., Goto H. (2010). Histological intestinal alternations in chickens fed low protein diet. J. Anim. Physiol. Anim. Nutr..

[57] Weiser M.M. (1973). Intestinal epithelial cell surface membrane glycoprotein synthesis. I: An indicator of cellular differentiation. J. Biol. Chem..

[58] Ferraris R.P., Villenas S.A., Diamond J.M. (1992). Regulation of brush border enzyme activities and enterocyte migration in mouse small intestine. Am. J. Physiol..

[59] Dong X.Y., Wang Y.M., Dai L., Azzam M.M.M., Wang C., Zou X.T. (2012). Posthatch development of intestinal morphology and digestive enzyme activities in domestic pigeons (*Columba livia*). Poult. Sci..

